# Pre-existing Immunocompromised Status as a Preventer of Mortality in COVID-19 Patients: Friend or Foe?

**DOI:** 10.7759/cureus.37633

**Published:** 2023-04-16

**Authors:** Sehnaz Olgun Yıldızeli, Duygu Vezir, Canan Cimsit, Derya Kocakaya, Zeynep Mercanci, Baran Balcan, Onur Ermerak, Can Ilgin, Emel Eryuksel, Sait Karakurt

**Affiliations:** 1 Pulmonary and Critical Care Medicine, Marmara University School of Medicine, Istanbul, TUR; 2 Radiology, Marmara University School of Medicine, Istanbul, TUR; 3 Thoracic Surgery, Marmara University School of Medicine, Istanbul, TUR; 4 Public Health, Marmara University School of Medicine, Istanbul, TUR

**Keywords:** mortality, immunocompetent, immunosuppressed, pneumonia, covid-19

## Abstract

Objective

COVID-19 has been negatively impacted by a number of comorbidities. Aside from that, some conditions or treatments that cause immunosuppression can alter the course of the disease, leading to worse outcomes. The primary goal of this study is to compare the clinical presentation, laboratory analysis, radiological findings, and outcomes of patients with COVID-19 with and without immunosuppression.

Materials and methods

The study includes patients with pre-existing immunosuppression and COVID-19 infection who were admitted and received inpatient treatment at Marmara University Hospital, Istanbul, Pulmonary Medicine ward between April 2020 and June 2020. Data on demographics, epidemiology, clinical course, laboratory analysis, radiological findings, length of hospital stay, morbidity, and mortality were collected from all patients.

Results

The study group consisted of 23 patients who had pre-existing immunosuppression, and the control group consisted of 207 immunocompetent patients, making a total of 230 patients. Significant differences in lymphocyte count, ROX (respiratory-rate oxygenation) index on Day 0, and fibrinogen levels were discovered between the two groups. SARI (severe acute respiratory infection) was more common in the control group than in the study group (p<0.022), but there was no difference in mortality.

Conclusion

The mean number and percentage of lymphocytes were lower in immunocompromised COVID-19 patients at the time of diagnosis. Higher ROX index values and a lower risk of developing SARI could explain the hypothesis that these patients may be benefiting from a pre-existing corticosteroid regimen. Additional research with larger numbers of patients may be beneficial in drawing a more definitive conclusion.

## Introduction

The novel coronavirus disease (COVID-19) first emerged in Wuhan, China in December 2019 and quickly spread throughout the world, becoming a pandemic. Despite the fact that COVID-19 primarily affects the respiratory system, it can present in a variety of clinical pictures due to its ability to infect various organs and tissues [1]. According to World Health Organization (WHO) data, 664 million patients were infected with the virus, with 6.7 million of them dying to date [2]. Following the rapid onset of the epidemic, numerous studies on the demographics, epidemiology, clinical presentation, and radiological and laboratory findings of the disease were conducted. These studies provided critical information about the treatment options and prognosis of the disease [3]. Age, male gender and the presence of specific comorbidities such as diabetes, obesity, coronary artery disease (CAD), chronic lung disease, and chronic renal failure (CRF) have been shown to have a negative impact on respiratory complications and mortality [4]. Furthermore, the importance of both innate and adaptive immunity in controlling viral infection has been emphasized, and the majority of this data came from studies on immunocompetent patients with COVID-19 infection. Unfortunately, a significant number of patients with immunocompromised status due to pre-existing conditions cannot tolerate discontinuing immunosuppressive therapy under any circumstances. Although the level of immunosuppression varies depending on the regimen, this specific group of patients is known to be predisposed to opportunistic viral infections [5]. Thus, increased mortality, morbidity, and hospitalization were anticipated in these immunocompromised patients infected with COVID-19 [6]. There have been a few publications in the literature about this specific group of patients, reported mostly by rheumatologists and gastroenterologists. Unfortunately, the small number of studies available does not provide enough information to conclude the entire course and prognosis [7-9].

The primary aim of this study is to review the inpatient course of patients with COVID-19 infection who are already immunocompromised due to underlying disease and/or treatment and report the clinical presentation, laboratory analysis, radiological findings, and outcomes. Furthermore, the results will be compared with data from studies on immunocompetent patients with COVID-19 infection.

## Materials and methods

The study was designed as a retrospective cohort study. The study included patients with pre-existing immunosuppression and COVID-19 infection who were admitted and receivedinpatient treatment between April 2020 and June 2020. Data on demographics, epidemiology, clinical course, laboratory analysis, radiological findings, length of hospital stay, morbidity, and mortality of patients were extracted from our University Hospital's electronic medical records (EMR) and systematically entered into a database. The study was approved by Marmara University Research Ethics Board (REB).

Patient selection

Between April 2020 and June 2020, all patients aged 18 and above who were admitted with a positive reverse transcription polymerase chain reaction (RT-PCR) nasopharyngeal swab for COVID-19 were analyzed. Those with pre-existing immunosuppression and COVID-19 infection are enrolled in the study. This mostly included patients with a history of solid organ or bone marrow transplantation, who have been treated with chemotherapy for any type of malignancy, any biological agent, prednisone equivalent of more than 10 mg/day and 700 mg cumulative dose [10], antiproliferative agents and B-/T-cell depletion medications. The control group, on the other hand, consisted of the immunocompetent patients who were admitted with a positive RT-PCR nasopharyngeal swab for COVID-19 during the same time interval.

Clinical presentation and laboratory analysis

Symptoms, the time interval between the first symptom and diagnosis, radiological findings, length of stay in the Severe Acute Respiratory Infections (SARI) Unit or Intensive Care Unit (ICU), medical treatment received during admission, and the length of total hospital stay were all reviewed and recorded systematically. SARI is an acute respiratory tract infection that occurred within the previous 14 days and necessitated hospitalization due to fever, cough, dyspnea, tachypnea, hypoxemia, hypotension, diffuse radiological pathological findings, or a decrease in the level of consciousness [11]. Need for high oxygen support (>15 lt/min), hemodynamic instability, and decrease in the level of consciousness were used as criteria for admission to ICU and patients with one or more of these criteria were admitted to the ICU. Patients who required extracorporeal membrane oxygenation (ECMO) treatment were also explicitly identified and recorded. The national guidelines of the Turkish Ministry of Health, which were created and updated in light of new developments and current international guidelines at the time of the study, were followed while arranging the patient’s treatment regimens. The main parameters that we looked at in the follow-up period were the level and percentage of lymphocytes on a complete blood count with differential, as well as the level of C-reactive protein (CRP), ferritin, pro-brain natriuretic peptide (pro-BNP), fibrinogen, activated partial thromboplastin time (aPTT), and D-dimer. Furthermore, from the first post-admission day (PAD 0) to the last day of hospitalization, the respiratory-rate oxygenation (ROX) index was calculated for each patient daily.

Radiological evaluation

The patients’ radiological images were independently interpreted by two experienced thoracic radiologists, and the results were reported in accordance with the American College of Radiology-endorsed Expert Consensus Statement on reporting chest imaging findings related to COVID-19. Some specific criteria were considered to reach a standardized conclusion. These criteria are as follows: 1) distribution: peripheral or peribronchovascular; 2) density: ground glass, consolidation or mixed pattern; 3) internal structures: the presence of air bronchogram, interlobular septal thickening, and cavitation; 4) the number of lobes affected: ground glass or nodular pattern; 5) the presence of fibrotic lesions; 6) centrilobular opacities; 7) the presence of pleural effusion; 8) the presence of lymphadenopathy (short axis of 10 mm and above); 9) findings related to an underlying disease causing parenchymal pathology (emphysema, tuberculosis, etc.). The total severity index was calculated on a scale of 0 to 20 by evaluating the involvement of each lobe separately. The degree of involvement was classified as follows: no involvement (0), minimal (1-25%), mild (26-50%), moderate (51-75%), and severe (76-100%), with each category receiving a score ranging from 1 to 4 respectively [12].

Statistical analysis

Stata 15.1 software (StataCorp, College Station, USA) was used to perform all statistical analyses. Numerical variables were reported as either mean±standard deviation or median and interquartile range (IQR) including minimum and maximum values, according to their distributions. The normality assumption was tested with histogram and normal quantile plots, Skewness Kurtosis, and Kolmogorov-Smirnov tests. The categorical variables were reported with frequencies and percentages. The comparisons of the distributions of numerical variables among two independent groups were analyzed by using the Mann-Whitney U test. The cross tables of categorical variables were analyzed with Chi-Square or Fisher’s exact tests. A p-value less than 0.05 was considered significant. The power of the study calculated with the current number of patients was 70%.

## Results

The files of 279 patients with COVID-19 infection who were admitted and received inpatient treatment between April 2020 and June 2020 were reviewed and 230 of them were found eligible to be enrolled because their files did not have any information required for the study missing.

Demographic features

The study group included 23 of the 230 patients who had pre-existing immunosuppression, while the remaining 207 patients were classified as the control group defined as immunocompetent. The study group has a female-to-male ratio of 11/12 with a mean age of 52.8±12.8 years while the control group has a female-to-male ratio of 91/116 with a mean age of 55±15.7 years (p>0.05).

As previously stated, immunosuppression occurs as a result of medications administered to treat pre-existing conditions. These situations included solid organ transplantation in three patients (13%), hematological malignancy in three patients (13%), ongoing chemotherapy in five patients (21.7%), biological agent (adalimumab) treatment in two patients (8.7%), steroid therapy in six patients (26%), anti-proliferative therapy in seven patients (30.4%), treatment with calcineurin inhibitors in three patients (13%), and use of agents acting on T cells in eight patients (34.7%) and on both T and B cells in 12 patients (52%) (Table 1).

**Table 1:** Baseline characteristics of immunosuppressed patients

Age (years) mean range	52.78+12.79
Female n (%)	11 (43.5%)
Solid organ transplantation (%)	3 (13%)
Hematological malignancy	3 (13%)
Chemotherapy	5 (21.7%)
Biological agent therapy	2 (8.7%)
Systemic Corticosteroids	6 (26%)
Antiproliferative drugs	7 (30.4%)
Calcineurin inhibitors	3 (13%)
Drugs affecting T and B cells	12 (52 %)
Drugs affecting T cells	8 (34.7%)

Comorbidities

**Figure 1:**
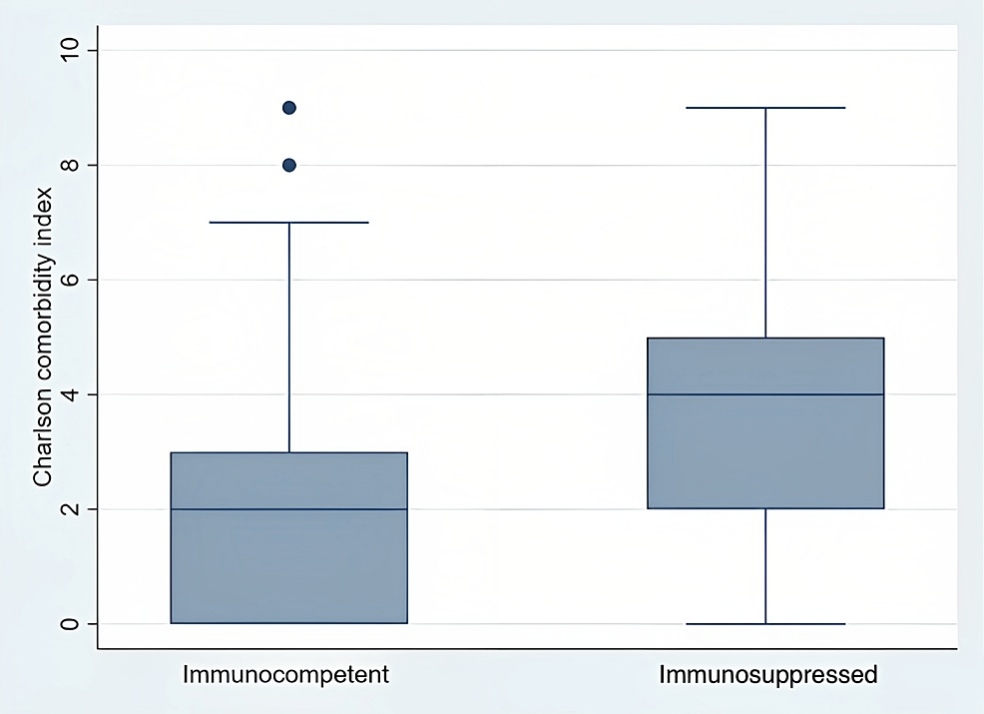
Charlson comorbidity index for immunosuppressed and immunocompetent patients

The median Charlson Comorbidity Index was 4 (IQR: 0-9) in the study group while it was 2 (IQR: 0-7) in the control group which included immunocompetent patients (Figure 1). Comorbidities of the study group were as follows: hypertension in six patients (26%), cardiac disease in one patient (4.4%), chronic respiratory disease in three patients (13%), diabetes mellitus in four patients (17.4%), inflammatory bowel disease in one patient (4.4%), thyroid function abnormalities in four patients (17.4%), dyslipidemia in one patient (4.4%), dementia in one patient (4.4), various types of malignancies in 11 patients (47.8%) and rheumatological disease in seven patients (30.4%). On the other hand, comorbidities of the control group were; hypertension in 77 patients (37%), cardiac disease in 36 patients (17.3%), a chronic respiratory condition in 27 patients (13%), diabetes mellitus type 2 in 64 patients (30.7%), thyroid function abnormalities in six patients (2.3%), dyslipidemia in seven patients (3.4%), various types of malignancies in five patients (2.4%), and rheumatological disease in three patients (3.4%). There were statistically significant differences between groups in terms of malignancies (p<0.0001), rheumatologic diseases (p<0.001), and thyroid function abnormalities (p<0.011) (Table 2).

**Table 2:** Distribution of the comorbidities in both groups

	Immunosuppressed n (%)	Immunocompetent n (%)	p-value
Hypertension n (%)	6 (26%)	77 (37%)	0.23
Cardiac disease n (%)	1(4.4%)	36 (17.3%)	0.13
Chronic respiratory system disease n (%)	3(13%)	27 (13%)	1.00
Diabetes mellitus n (%)	4 (17.4%)	64 (30.7%)	0.23
Inflammatory bowel disease n (%)	1 (4.4%)	0	1.00
Hyper-hypothyroidism n (%)	4 (17.4%)	6 (2.3%)	0,011
Hyperlipidemia n (%)	1 (4.4%)	7 (3.4%)	0.5
Dementia n (%)	1 (4.4%)	0	1.00
Malignancy n (%)	11 (47.8%)	5 (2.4%)	<0.0001
Rheumatological diseases n (%)	7 (30.4%)	3 (1.4%)	<0.001

Symptoms

Fever, cough, shortness of breath (SOB), abdominal pain, nausea-vomiting, loss of sense of smell, myalgia, and fatigue were the most common symptoms. Rhinopharyngitis, loss of appetite, and diarrhea were only seen in immunocompetent patients, with three (1.4%), 13 (8.17%), and 17 (8.2%) cases, respectively. The symptoms were evaluated and compared between the two groups. The only significant difference in symptomatology was lassitude (Table 3). In addition to these findings, the time interval between the first symptom and diagnosis was found to be 3.6±3.7 days in immunocompromised patients (study group) and 4.8±5.7 days in the immunocompetent patients (control group) (p>0.5).

**Table 3:** COVID-19-related symptoms

	Immunosuppressive n:23 (%)	Immunocompetent n:207 (%)	p-value
Fever	8 (34.8 %)	77 (31.2%)	0.807
Cough	13 (56.5%)	116 (56%)	0.965
Dyspnea	10 (43.5%)	83 (40.1%)	0.768
Rhino-pharyngitis	0	3 (1.4%)	1.00
Dysosmia-dysgeusia	1 (2.3%)	6 (2.9%)	0.728
Diarrhea	0	17 (8.2%)	0.23
Abdominal pain	1 (2.3%)	6 (2.9%)	0.528
Nausea- vomiting	1 (2.3%)	25 (12.1%)	0.48
Loss of appetite	0	13 (8.17%)	1.00
Myalgia	5 (21.8%)	17 (8.21%)	0.054
Lassitude	7 (30.4%)	20 (9.7%)	0.013

Clinical staging

Patients in both groups were evaluated using a clinical scoring system based on the time course and severity of illness (Stage I for early infection, Stage II for pulmonary phase, and Stage III for hyperinflammatory phase) [13] and classified based on their stage at the time of admission. All of the 11 patients (5.3%) in the Stage I group were from the control group. Stage IIa included 20 patients (87%) from the study group and 130 patients (62.3%) from the control group while Stage IIb included seven patients (30.4%) from the study group and 75 patients (36%) from the control group. Additionally, three patients (13%) were from the study group and 32 patients (15.3%) were from the control group in Stage III. There was no significant difference between groups in terms of clinical staging.

Laboratory analysis

The results of the laboratory tests were recorded during the admission, beginning on the first day of hospitalization (Day 0). The lowest values were taken into consideration for the count and percentage of the lymphocytes, as well as the ROX index. In addition to that, values for CRP, ferritin, pro-BNP, fibrinogen, aPTT, and D-dimer were recorded daily. Significant differences in lymphocyte count, ROX index on Day 0, and fibrinogen during admission were found between the two groups (Table 4).** **

**Table 4:** Laboratory findings of both groups CRP: C-reactive protein; ROX: respiratory-rate oxygenation

	Immunosuppressive	Immunocompetent	*p*-value
Lymphocyte count day 0 Median (p50)	1000 (200-2100) IQR: 1000	1100 (100-4500) IQR: 800	0.022
Lymphocyte count min during hospitalization Median (p50)	600 (200-2100) IQR: 600	900 (0-3100) IQR: 800	0.114
CRP day 0 Median (p50)	29 (3-258) IQR: 62.9	26.7 (3-329) IQR: 61	0.747
CRP max during hospitalization Median (p50)	77.5 (3-280) IQR: 91.4	58.4 (3-427) IQR: 125.6	0.966
Ferritin day 0 Median (p50)	207 (5.4-2770) IQR: 362	164.3 (2.7-2845) IQR: 341.35	0.380
Ferritin max during hospitalization Median (p50)	338 (6.4-1500) IQR: 666	293 (2.7-1550) IQR: 556	0.651
Fibrinogen day 0 Median (p50)	430 (91-767) IQR:247	442 (103-899) IQR: 167	0.482
Fibrinogen max during hospitalization Median (p50)	423 (51-1161) IQR: 239	526 (41-1037) IQR: 261.5	0.015
D-dimer day 0 Median (p50)	0.89 (0.26-3.65) IQR: 1.14	0.58 (0.14-20) IQR: 0.57	0.118
D-dimer max during hospitalization	1.12 (0.31-10.86) IQR: 3.2	0.83 (0.14-20) IQR: 1.56	0.373
ROX index day 0 Median (p50)	23.3 (4.46-28.5) IQR: 6.3	19.6 (2.25-33.3) IQR: 8.6	0.003
ROX index min during hospitalization Median (p50)	19.9 (2-26.19) IQR: 9.35	14.96 (1.64-29.16) IQR: 13.2	0.066

Medical treatment

The mainstay of medical treatment was carried out following the guidelines of the Turkish Ministry of Health, in light of the data derived from international recommendations. The following medications are included in this guideline: hydroxychloroquine sulfate, azithromycin, favipiravir, tocilizumab, convalescent plasma therapy, and corticosteroids. The majority of patients in both groups received hydroxychloroquine sulfate as treatment, with 22 patients (95.7%) in the study group and 207 patients (99.5%) in the control group (p>0.19). Azithromycin was preferred in eight patients (37.8%) in the study group and 81 patients (38%) in the control group (p>0.69) while favipiravir was the treatment of choice in 10 patients (43.5%) in the study group and 83 patients (40%) in the control group (p>0.74). While 17 patients (8.17%) from the control group were treated with tocilizumab, only one patient (4.35%) received it from the study group (p>0.41). Similarly, only one patient (4.35%) used corticosteroids from the study group compared to 19 patients (9.13%) from the study group (p>0.70). Furthermore, only one patient from each group received convalescent plasma therapy with a percentage of 4.35 and 0.5, respectively (p>0.19). There was no statistically significant difference between groups in terms of medical treatment regimens used.

Radiological findings

**Figure 2:**
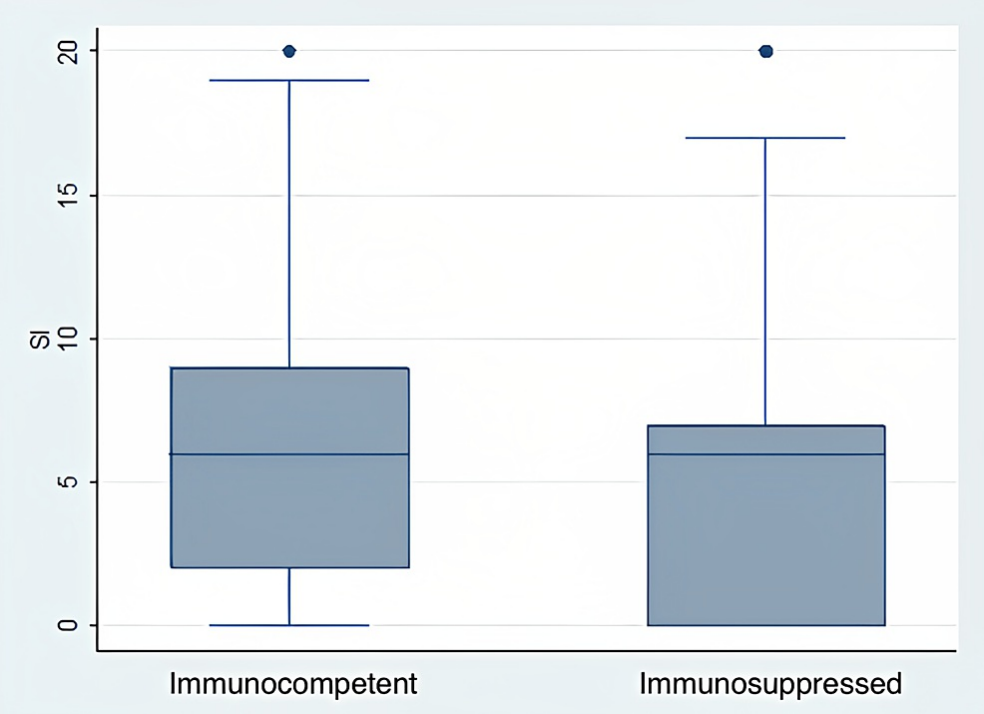
Severity indexes of immunosuppressed and immunocompetent patients

The median severity score for the computed tomography (CT) scan of the chest was calculated as 6 (0-20 IQR:7) for both groups (p=0.56) (Table 5, Figure 2). The most common imaging findings are pleural effusion, lymphadenopathy (LAN), subpleural ground glass opacity (GGO), irregular consolidation, and fibrosis. When we compared the numbers and percentages of the findings between the study and control group, we saw the presence of pleural effusion in three patients (13.7%) vs 31 patients (15.9%) (p>0.71), LAN in six patients (27.2%) vs 42 patients (21.4%) (p>0.53), subpleural GGO in 15 patients (68.1%) vs 152 patients (77.5%) (p>0.32), irregular consolidation in five patients (22.8%) vs 66 patients (33.9%) (p>0.29) and fibrosis in three patients (13.7%) vs 38 patients (19.6%) (p>0.5) between the study group and the control group. Radiologically, there was no significant difference in any parameter between the two groups.

↵

**Table 5:** Radiological findings for both groups

	Immunosuppressive	Immunocompetent	*p*-value
Severity index median (min-max)	6 (0-20) IQR: 7	6 (0-20) IQR:7	0.56
Pleural effusion n (%)	3 (13.7%)	31 (15.9%)	0.71
Lymphadenopathy n (%)	6 (27.2%)	42 (21.4%)	0.53
Subpleural ground glass n (%)	15 (68.1%)	152 (77.5%)	0.32
Large irregular consolidation n (%)	5 (22.8%)	66 (33.9%)	0.29
Fibrosis n (%)	3 (13.7%)	38 (19.6%)	0.50

Clinical course and mortality

Among the 23 immunocompromised patients, six (26%) were affected by SARI and three (13%) required ICU admission. None of the patients in the study group required ECMO during the follow-up period of hospitalization. On the other hand, 106 patients (51.2%) in the control group developed SARI, 41 (19/9%) required ICU admission, and two (0.97%) required treatment with ECMO. SARI was found to be more frequent in the control group than in the study group, with a significantly higher rate of development (p<0.022). In terms of mortality, three patients (13%) died in the study group, while 19 patients (9.18%) were pronounced dead in the control group (p>0.46) (Table 6). All three patients who died in the study group were actively receiving chemotherapy for esophageal cancer, metastatic lung cancer, and multiple myeloma.

**Table 6:** Clinical deterioration during hospitalization and mortality * ICU: Intensive care unit, ** SARI: severe acute respiratory infections, *** ECMO: extracorporeal membrane oxygenation

	Immunosuppressive	Immunocompetent	p-value
Mortality	3 (13.04%)	19 (9.18%)	0.463
ICU ^*^	3 (13.04%)	41 (19.9%)	0.581
SARI ^**^	6 (26.09%)	106 (51.2%)	0.022
ECMO ^***^	0	2 (0.97%)	1.00

## Discussion

Although high mortality rate is thought to be related to the negative impact of the virus on the immune system of the patient, the exact pathophysiology is still unknown. Moreover, it is well-known that immunocompromised patients are predisposed to opportunistic infections as a result of immune system impairment. Unfortunately, data regarding the co-occurrence of immunosuppression and COVID-19 infection is limited and based solely on case reports. Thus, we designed this study to examine the inpatient course of patients with COVID-19 infection who have a prior immunocompromised status due to an underlying disease. Furthermore, it is intended to create a cohort to compare the results with COVID-19 patients who have a competent immune system.

We retrospectively reviewed the data of 23 COVID-19 patients who had pre-existing immunosuppression, and 207 controls with immunocompetent status. When we looked back at our approach at the start of the pandemic, we expected patients with pre-existing immunosuppression to have higher mortality rates related to a worse prognosis with more rapid functional deterioration. As a result of broadening the indications and keeping the threshold lower for the decision of admission, the majority of those patients were hospitalized earlier.

The most common symptoms can be summarized as fever, cough, SOB, abdominal pain, nausea-vomiting, loss of sense of smell, myalgia, and fatigue. Even though we expect COVID-19 to have a more severe clinical course in immunocompromised patients, only fatigue and myalgia were found to be significantly higher in the study group compared to the control group in our cohort. In close similarity with our data, Bezzio et al. published a study regarding the symptomatology of COVID-19 patients with pre-existing inflammatory bowel diseases and found no significant difference in terms of symptoms except diarrhea when compared to patients with a competent immune system [9]. Furthermore, Monti and colleagues reported a case series involving 13 patients whose immune systems were compromised as a result of medications used for rheumatological diseases. This study showed that symptoms and severity of COVID-19 did not show any significant difference compared to patients with COVID-19 and healthy immune systems [14].

Although the time interval from the first symptom to the diagnosis of COVID-19 was not different between immunocompromised and immunocompetent patients [15], our study found that this interval was shorter in immunocompromised patients but not statistically significant when compared to the control group. This shorter interval can be explained most effectively by the fact that symptoms in immunocompromised patients are taken more seriously by both the patient and the physician. However, it was found that the distribution of the clinical stages differs between the two groups. Even though patients in the control group were evenly distributed across stages, all patients in the study group were assigned to either Stage IIa or IIb. There was no difference between groups in terms of worse prognosis when we used the worst stage of each patient as a parameter for comparing the clinical deterioration and worse prognosis. Although our findings are consistent with those of D’Antiga [4] and Hui et al [16], Zhu et al [17] stated otherwise in a publication showing a higher rate of clinical deterioration in immunocompromised patients. This disparity in results could be attributed to the diversity in immunosuppressive regimens, which are tailored to the type and severity of the underlying disease. Therefore, patients in Zhu et al’s study were post-transplant patients and they had received an immune suppressive regimen including triple medications whereas patients in the other studies mentioned above had received immunosuppressive therapy with single or dual medications.

In the study group, lymphocyte count/percentage and fibrinogen levels were found significantly lower on the first day of the hospitalization. Additionally, the values for the ROX index were higher in the study group than in the control group. Although no publication exists in the literature analyzing the laboratory parameters of this specific group of patients with pre-existing immunocompromised status, only Zhu and colleagues mentioned significant lymphopenia in renal transplant patients on the day of admission [17]. Our findings corroborated Zhu et al’s findings, and we believe that this condition is related to the intensity of the immunosuppressive regimen of the transplantation patients. The ROX index is primarily used to predict high-risk patients who will rapidly deteriorate due to an increased risk of intubation [18]. The ROX index values in our cohort were higher during and on the day of admission in the study group, but the results did not show a significant difference.

Despite the lack of refined data focusing specifically on patients with pre-existing immunosuppression, only a few reports concluded that immunosuppression secondary to malignancy, autoimmune disease, or organ transplantation does not increase the risk of respiratory complications and mortality [4, 16]. In contrast to these few publications, only Zhu et al compared transplanted patients to otherwise healthy patients with COVID-19 in their close families. Even though they did not use the ROX index as a parameter in their evaluation, they identified immunosuppression as an independent risk factor for severe pneumonia. On the other hand, our cohort surprisingly revealed a higher rate of SARI development in the control group, which we believe is due to the early hospitalization of these patients with immunosuppression because of their alerted awareness of having a serious chronic condition that is under ongoing surveillance by a group of dedicated physicians. Moreover, some of those patients were already receiving corticosteroid therapy as part of their pre-existing treatments when they became infected with COVID-19. Corticosteroids were not included in our national protocol for the management of COVID-19 at the time of this study because there was insufficient scientific evidence for the positive role of corticosteroid use in the treatment of COVID-19. As a result, we believe that higher ROX index values and a lower risk of developing SARI in the control group are related to corticosteroid therapy.

In terms of radiological analysis, there was no difference between the study and control groups in any given capacity. In addition, there was no statistically significant difference between mortality or the need for ICU admission between groups. These findings are consistent with previous research conducted on a similar group of patients [4, 16]. In contrast to data from these few publications, Zhu and colleagues reported faster clinical deterioration and prolonged viral eradication in this specific group of patients, though their findings showed that the rate of unfavorable fatal outcomes was reduced due to suppression of hyperimmune response. The increased mortality of COVID-19 is solely related to acute respiratory distress syndrome (ARDS) developing as a result of the hyperimmune response, including cytokine storm, as seen in the second phase of the disease. This lowered hyperimmune response is thought to explain why COVID-19 mortality rates are lower in this immunocompromised group of patients [17]. Although the results of our study are based on a limited number of patient results, they also support the association of lower lymphocyte counts and less severe clinical presentation with reduced hyperimmune response in immunosuppressed patient groups.

These results show us that patients with previous immunosuppression will require close follow-up, but may also provide clinical benefit in terms of avoiding an aggressive approach in terms of intensive care follow-ups.

There are some limitations to this study. Because of the retrospective design of the study, some data may be missing or limitations in the evaluation may have occurred. Due to the small number of immunocompromised patients, subgroup analysis was not possible. Another point to consider is that the study is based on the data from the first attack of the pandemic in our country, and the lack of clinical experience with COVID-19 at the time created a bias because the majority of the immunocompromised patients were admitted to the hospital earlier as a precaution as opposed to patients with a competent immune system.

## Conclusions

In conclusion, we discovered that the mean number and percentage of lymphocytes were lower at the time of the diagnosis in COVID-19 patients who had pre-existing immunosuppression compared to COVID-19 patients with competent immune status. Higher ROX index values and a lower likelihood of developing SARI may support the idea that pre-existing immunosuppressive states have a positive impact on these patients. More studies involving more patients may be beneficial in drawing a more definitive conclusion about these issues.
